# The Two Sides of Complement C3d: Evolution of Electrostatics in a Link between Innate and Adaptive Immunity

**DOI:** 10.1371/journal.pcbi.1002840

**Published:** 2012-12-27

**Authors:** Chris A. Kieslich, Dimitrios Morikis

**Affiliations:** Department of Bioengineering, University of California, Riverside, Riverside, California, United States of America; University of California San Diego, United States of America

## Abstract

The interaction between complement fragment C3d and complement receptor 2 (CR2) is a key aspect of complement immune system activation, and is a component in a link between innate and adaptive immunities. The complement immune system is an ancient mechanism for defense, and can be found in species that have been on Earth for the last 600 million years. However, the link between the complement system and adaptive immunity, which is formed through the association of the B-cell co-receptor complex, including the C3d-CR2 interaction, is a much more recent adaptation. Human C3d and CR2 have net charges of −1 and +7 respectively, and are believed to have evolved favoring the role of electrostatics in their functions. To investigate the role of electrostatics in the function and evolution of human C3d and CR2, we have applied electrostatic similarity methods to identify regions of evolutionarily conserved electrostatic potential based on 24 homologues of complement C3d and 4 homologues of CR2. We also examine the effects of structural perturbation, as introduced through molecular dynamics and mutations, on spatial distributions of electrostatic potential to identify perturbation resistant regions, generated by so-called electrostatic “hot-spots”. Distributions of electrostatic similarity based on families of perturbed structures illustrate the presence of electrostatic “hot-spots” at the two functional sites of C3d, while the surface of CR2 lacks electrostatic “hot-spots” despite its excessively positive nature. We propose that the electrostatic “hot-spots” of C3d have evolved to optimize its dual-functionality (covalently attaching to pathogen surfaces and interaction with CR2), which are both necessary for the formation B-cell co-receptor complexes. Comparison of the perturbation resistance of the electrostatic character of the homologues of C3d suggests that there was an emergence of a new role of electrostatics, and a transition in the function of C3d, after the divergence of jawless fish.

## Author Summary

Complement fragment C3d is a thioester-containing protein that is a key component/domain in the complement system, an ancient line of defense, due to its ability to covalently attach to pathogen cell surfaces, such as bacteria. As the immune system evolved in complexity, from acellular defense mechanisms to multicellular systems with memory, so has the function of C3d. In humans, but not lower species such as invertebrates, C3d attached to pathogen surfaces binds B-cell co-receptor CR2, in conjunction with an antibody/antigen complex, forming a link between the innate and adaptive immune systems. The C3d-CR2 interaction ultimately increases B-cell sensitivity to the C3d tagged pathogen by 1,000–10,000 fold, and is known to be driven by electrostatic forces. Since electrostatics are crucial to the C3d-CR2 interaction, it is likely that probing the evolution of the electrostatics of C3d and CR2 will provide insight into this gained function. To this end, we employ a novel computational approach for identifying the electrostatic “hot-spots” of C3d and CR2, which are produced by clusters of like-charged residues found on the surface of the protein. Electrostatic “hot-spots” are often evolutionarily favored and in this study provide new insight into the evolution of C3d in its role in a link between innate and adaptive immunity.

## Introduction

**Figure 1:**
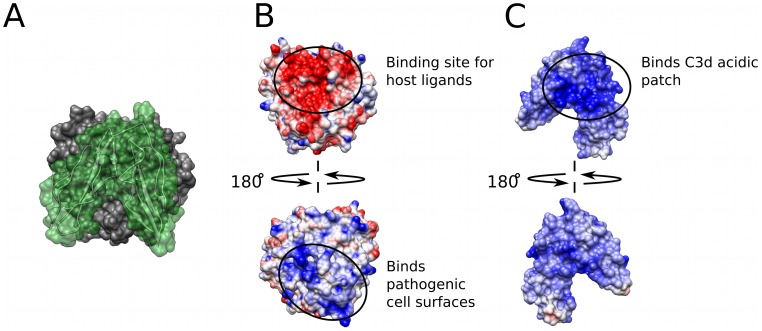
Molecular representations of the C3d-CR2 interaction. (A) Surface representation of the C3d-CR2 interaction with C3d in gray and CR2 in green (PDB Code: 3OED). (B) Electrostatic potential surface projection for C3d (PDB Code: 1C3D). (C) Electrostatic potential surface projection for CR2 (PDB Code: 1LY2). The code for panels (B) and (C) are as follows: the color transitions from red – white – blue when going from negative (−5 kT/e) – neutral (0 kT/e) – positive (+5 kT/e) electrostatic potential.

The complement immune system is a vital component of innate immunity that attacks foreign pathogens by covalently attaching to pathogen antigens, directly lysing pathogen surface membranes, and initiating inflammatory responses. In humans, one key result of complement activation is the formation of B-cell co-receptor complexes, which form a link between innate and adaptive immunities, and increase B-cell sensitivity to an antigen by 1000–10000 fold [1]–[4]. During B-cell co-receptor complex formation, a critical interaction must occur between complement fragment C3d, and the first two complement control protein domains of complement receptor 2, CR2(CCP1-2) [referred henceforth as simply CR2] (Figure 1A) [1]–[3]. Complement C3d is a domain and cleavage product of complement protein C3, the central protein involved in the activation and up regulation of the complement immune system, while CR2 is a membrane protein that is expressed on the surface of B-cells [1], [2]. As a domain of complement C3, C3d is involved in the opsonization of pathogens through a highly reactive thioester bond, which can ultimately result in pathogens being coated by covalently attached C3d [1], [2]. Evolutionarily, C3d is of great interest since C3 can be found in species that have been on the earth for 600 million years, while CR2 and the C3d-CR2 interaction is believed to have been gained much more recently after the appearance of adaptive immunity, which first appeared in teleost fish [4]. Therefore, C3d has evolved to be dual-functional, since C3d covalent attachment to pathogen cell surfaces and the C3d-CR2 interaction must both occur simultaneously in order to form B-cell co-receptor complexes. On the other hand, CR2(CCP1-2) is believed to be mono-functional, with its interaction with C3d being its sole function.

Due to the significance of the C3d-CR2 interaction and its role in increasing B-cell sensitivity, extensive research has been performed investigating the nature of the interaction [5]–[12], as well as possible approaches for utilizing this interaction in the design of new therapeutics and vaccines [1], [13]. As has been discussed for many complement protein interactions, electrostatic forces contribute significantly to the C3d-CR2 interaction [11], [12]. Often as a result of evolution many proteins, especially those of the complement system, contain clusters of like-charged residues, which generate regions of high electrostatic potential that are often referred as electrostatic “hot-spots” [14], [15]. These electrostatic “hot-spots” tend to correspond with functional sites, since they can result in acceleration of protein association, and can stabilize protein complexes [16], [17]. This definition of electrostatic “hot-spots” differs from the typical definition of protein “hot-spots”, which refers to a residue that when mutated results in greater than 1 kcal/mol change in binding affinity [14], [15], since our definition involves the electrostatic contributions of numerous residues and can be understood in the absence of a protein complex.

When referring to the functional sites of C3d, two opposing surfaces have been described: 1) CR2-face, a highly acidic concave surface known to be the binding site of several host/pathogenic ligands (Figure 1B, top; see also Supporting Figures S1 and S2); and 2) thioester-face, a basic surface surrounding the thioester bond utilized in covalent attachment to pathogen cell surfaces (Figure 1B, bottom). The acidic “patch” has been shown to be involved in recognition and binding during the association of C3d to CR2 [8], [10], as well as to bacterial inhibitors of the complement system [18]–[20]. The basic surface however, accelerates the covalent attachment of C3/C3d to pathogenic cell surfaces. In comparison, CR2 possesses a predominantly positive electrostatic potential (Figure 1C), with the C3d binding site of CR2 having the most positive potential (Figure 1C, top), which is complementary to the acidic CR2 binding site on C3d.

Since electrostatics has been shown to play such a key role in the dual functionality of C3d, we propose that the electrostatic character of C3d has evolved to allow for optimal performance of both functions simultaneously. This stems from the fact that electrostatic forces contribute significantly to the C3d-CR2 interaction, and to covalent attachment of C3d to pathogen cell surfaces, both of which must occur for this link between innate and adaptive immunities to be activated. In this study, we investigate the presence of electrostatic “hot-spots” on complement proteins C3d and CR2 using a novel computational method involving perturbation of electrostatic properties of proteins. Additionally we probe the evolution of the electrostatic character of C3d and CR2, through the use of homology modeling, to gain insight into the role of electrostatics in the gained C3d-CR2 interaction, as well as the surface of the conserved thioester bond.

## Results/Discussion

**Figure 2:**
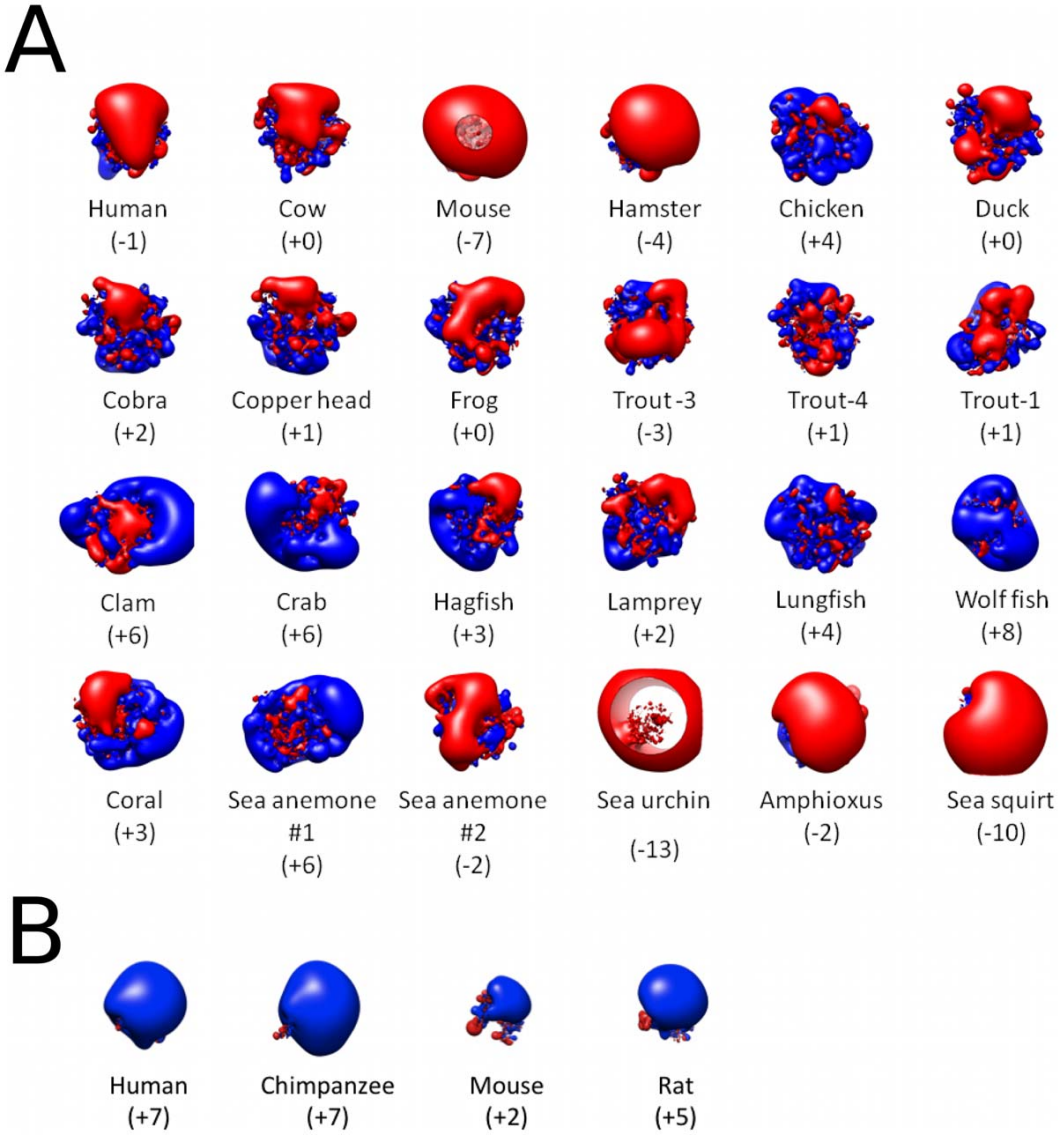
Spatial distributions of electrostatic potential for homologues of C3d and CR2. (A) Electrostatic potential distributions for 24 homologues of C3d [red, negative (−1.5 kT/e); blue, positive (1.5 kT/e)]. (B) Electrostatic potential distributions for 4 homologues of CR2 [red, negative (−1.5 kT/e); blue, positive (1.5 kT/e)]. The net charge (e) of each homologue is provided in the parentheses.

**Figure 3:**
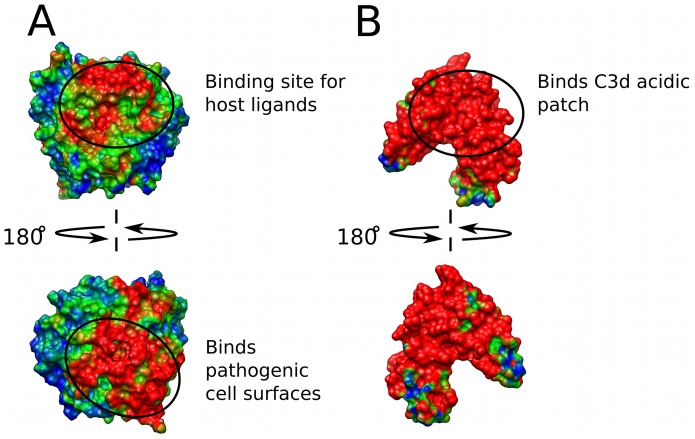
Conservation of electrostatic potential for homologues of C3d and CR2. (A) Cumulative electrostatic similarity distribution for 24 homologues projected onto the surface of human C3d. (B) Cumulative electrostatic similarity distribution for 4 homologues projected onto the surface of human CR2. The color transitions from blue – green – red when going from low to high similarity, and corresponds to ESI values of −0.10–0.15–0.40.

Through the course of evolution, in addition to sequence, electrostatic character is also often conserved. Conserved electrostatic potential can be responsible for acceleration and strengthening of protein-protein association, and is therefore indicative of the location of functional sites. Wade et al. have proposed computational methods utilizing homology modeling and Poisson-Boltzmann electrostatic calculations to quantitatively identify regions of conserved electrostatic character [21], [22]. The approach calculates electrostatic similarity indices (ESI) to determine the cumulative spatial distribution of electrostatic similarity across a family of homologous proteins. For this study, we have identified 24 homologues of C3d and 4 homologues of CR2, and the isopotential contours for the corresponding electrostatic potentials are provided in Figure 2. The C3d homologues were chosen from a variety of species, at various evolutionary time points, and are diverse both in sequence (∼36–84% identity with human) and in net charge (−13 to +8). However, the 4 identified homologues of CR2 are much more similar (net charge (+2 to +7) and ∼54–92% identity with human), and to the best of our knowledge, represent all currently known sequences of CR2. ESI calculations, similar to those proposed by Wade et al., were performed for the C3d and CR2 homologues (Figure 2), and are illustrated by the ESI surface projections of Figure 3. Despite large variations in the electrostatic potentials of the C3d homologues, the analysis identified two regions of high electrostatic similarity (Figure 3A; circled and indicated in red), which corresponded to the two functional sites of C3d. The ESI distribution for the CR2 homologues illustrate a high level of overall conserved electrostatic potential, not limited to the known functional site, as is indicated by the predominantly red surface projections of Figure 3B. Conservation of the electrostatic potential surrounding the functional sites of C3d and CR2 further suggests a central role for electrostatics in their functions; however, conserved electrostatic potential alone is not necessarily indicative of the existence of electrostatic “hot-spots”.

**Figure 4:**
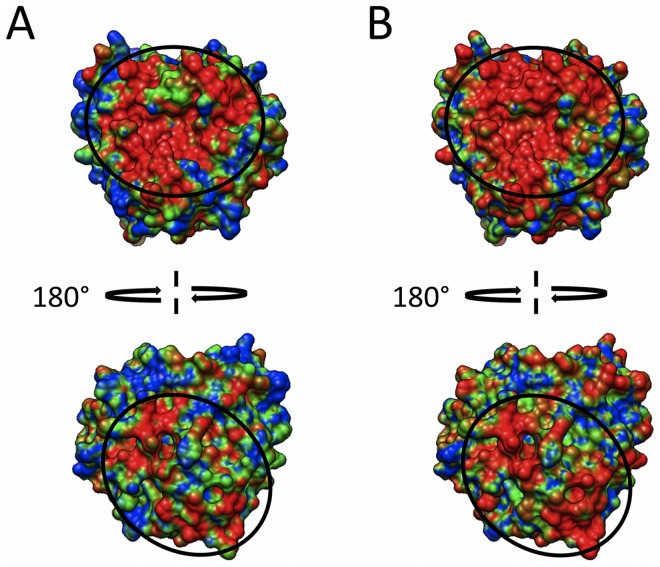
Electrostatic similarity distributions for perturbed human C3d structures. (A) Perturbation map for a 20 ns explicit-solvent MD simulation, based on 200 snapshots (extracted every 100 ps). (B) Perturbation map based on a theoretical alanine scan, consisting of 64 charged residue to alanine mutations. Color scheme is: blue – green – red; low to high similarity, corresponding to ESI values of 0.5–0.7–0.9.

If through the course of evolution, electrostatics has become crucial to protein function, it is plausible that the electrostatic nature of the protein would be resistant to perturbation. This stems from the assumption that a disruption in the protein electrostatic character would result in a reduction or loss of function. Furthermore, the necessity for electrostatic perturbation resistance is already suggested by the presence of clusters of like charged residues (“hot-spots”). To test this hypothesis, for the case of human C3d, we generated two sets of perturbed electrostatic potentials based on: i) molecular dynamics and ii) mutations. For the dynamics, a 20 ns explicit-solvent molecular dynamics (MD) simulation was performed, from which 200 conformations of C3d were extracted. As for the mutations, a computational alanine-scan was performed using the AESOP framework [23], in which each charged residue was mutated to alanine, one at a time. For both sets of perturbed structures, the procedure used to compare the C3d homologues was also applied to generate ESI distributions to identify regions of high electrostatic similarity, or those regions least affected by perturbation. Surface projections of the ESI distributions, referred henceforth as perturbation maps, were generated for the two sets of perturbed structures. Both perturbation maps, based on either dynamics or mutation, identify two similar regions with resistance to perturbation (Figure 4; circled and indicated in red). These regions correspond to the two functional sites of C3d (Figure 1B), as well as the two regions of evolutionarily conserved electrostatic potential (Figure 3A). This resistance to electrostatic perturbation of the two functional sites, suggest the presence on an electrostatic “hot-spot” at each site, which compensate for changes in the character of these regions. The two electrostatic “hot-spots” are slightly larger and more distinct in the mutation-based perturbation map (Figure 4B), when compared to the dynamics-based (Figure 4A), and this arises due to the size of the perturbation. This is understandable since depending on the amount of conformational change, dynamics can have noticeable effect on electrostatic potential; however, both perturbation types are still quite modest, yet are able to identify these electrostatic “hot-spots”.

An alanine-scan based perturbation map was also generated for human CR2 using the AESOP framework, and is illustrated by Figure S3. However, Figure S3A shows that no electrostatic “hot-spots” were identified on CR2 based on the same ranges of similarity used for C3d (Figure 4; 0.5–0.7–0.9), since the entire surface is colored blue indicating low electrostatic similarity. A second visualization of the CR2 alanine-scan perturbation map (Figure S3B), based on a much smaller range of similarities (0.47–0.48–0.49), still suggests a lack of distinct electrostatic “hot-spots” on the surface of CR2. Therefore, despite CR2 possessing excessive positive electrostatic potential, the charge residues of CR2 are evenly distributed and do not generate electrostatic “hot-spots”, such as those observed for C3d.

**Figure 5:**
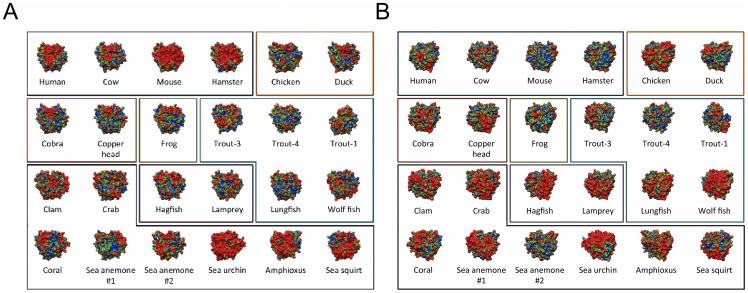
Mutation-based perturbation maps for 24 C3d homologues. The cumulative electrostatic similarity distributions for alanine-scan charge perturbations are projected onto the surface of each respective structure with two rotations: (A) CR2-face and (B) thioester-face. Perturbation map color scheme is: blue – green – red; low to high similarity, corresponding to ESI values of 0.5–0.7–0.9. Colored boxes are used to group homologues from similar species. The box color code is as follows: mammals, dark-blue; birds, orange; reptiles, red; amphibians, green; fish, light-blue; jawless fish, purple; invertebrates, black.

Given the presence of perturbation resistance in the electrostatic character of human C3d, the question of how exactly did this characteristic come about arises. This question is even more interesting, when considering that C3d has gained the CR2 interaction, which is driven by electrostatics, over the course of evolution. It is possible that either the C3d electrostatic “hot-spot” has always been present and CR2 was simply opportunistic, or that the “hot-spot” has come to existence through co-evolution with CR2, which seems the most likely. To investigate whether the C3d electrostatic “hot-spots” are present in homologues of human C3d, we generated perturbation maps based on alanine-scan mutations for the remaining 23 C3d homologues (Figure 5). When comparing the perturbation maps for the CR2-face of the C3d homologues (Figure 5A), we find that the mammals (dark-blue box) are the only group of species in which all homologues exhibit the CR2 “hot-spot”. This indicates that the electrostatic “hot-spot” of the CR2-face of C3d is something that has been gained through evolution. On the other hand, the electrostatic “hot-spot” on the thioester-face of C3d (Figure 5B) is much more predominant in lower species, such as the invertebrates (black box) and jawless fish (purple box), when compared to higher species like the mammals. The combination of these two results, the gain of the CR2 “hot-spot” and the reduction of thioester “hot-spot”, is quite interesting, since it suggests a transition in the function of C3d. The two functions of C3d can be seen as opposing one another, and in order to optimize the new interaction with CR2, the conserved electrostatic “hot-spot” on the thioester face was reduced or lost, such is the case for mouse. There are exceptions to the mentioned trends, mainly the invertebrates (Figure 5; black box), which exhibit large diversity in net charge (ranging from −13 to +6) and electrostatic potential (Figure 2). The invertebrates diverge first in the evolutionary tree, and therefore, have been evolving under their own pressures for much longer than any other group of species, which has most likely been the cause of this increased diversity. Interestingly, this increased diversity has resulted in the C3d of amphioxus having very similar electrostatic character to human C3d, when comparing net charge and electrostatic potentials/“hot-spots”, despite being separated by hundreds of millions of years in evolution. On a technical note, it should also be noted that due to the small size of the charge perturbations introduced by the alanine-scan mutations, electrostatic “hot-spots” could be overestimated in proteins with high net charge, which is most likely the case for homologues like the sea urchin (net charge −13).

**Figure 6:**
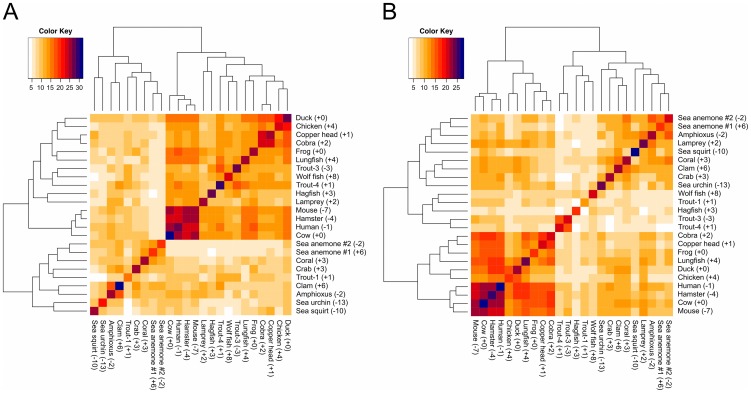
Charge similarity clustering for the two sides of C3d. Dendrograms with distance matrix heatmaps illustrate clustering of the 24 C3d homologues based on the number of positions with the same charge within the two functional regions: (A) CR2-face and (B) thioester-face. Net charge of each homologue is provided in parentheses.

Sequence-based approaches are typically used when analyzing the evolution of a protein, since conservation of amino acid positions can identify functionally important regions of sequence. As a comparison to our perturbation map approach, we have performed clustering for the 24 C3d homologues based on similarity of charged amino acid positions within the two functional regions (Figure 6). The CR2-face charge clustering (Figure 6A) identified two primary clusters: (1) contains all species with cellular immunity [jawless fish, fish, amphibians, reptiles, birds, and mammals]; (2) all invertebrate species. The appearance of jawless fish (lamprey and hagfish) in cluster 1 of the CR2-face clustering, is quite interesting given that jawless fish could hypothetically be the first group of species to exhibit a C3d-CR2-like interaction, since they contain B-like and T-like cells [24]. This is in contrast to the current hypothesis that teleost fish are the first species to possess the C3d-CR2 interaction [4]. The charge patterns of the species of cluster 1 are noticeably more similar (darker region; Figure 6A), suggesting an emergence of a new role of charge in the function of C3d in the species of this cluster. Additionally, the mammals cluster separately from the other species of cluster 1 in the CR2-face charge clustering. Indicating that the CR2-face charge character of mammal C3d homologues is unique, which correlates with the perturbation map results (Figure 5A). In contrast, the thioester-face charge similarity clustering (Figure 6B) produced a similar classification of the C3d sequences as found using sequence percent identity (Supporting Figures S4 and S5). It should be noted that the net charge of the homologues has little to no effect on the charge similarity clustering (Figure 6), and it's the position of specific charged residues that distinguishes the clusters of C3d homologues, which is in agreement with the existence of electrostatic “hot-spots”.

As has been discussed by McCammon [25], speed is often the main evolutionary driving force, even at the molecular level. Acceleration of biomolecular processes is achieved through long-range electrostatic interactions, which guide the formation of encounter complexes, increasing the diffusive rate [26]. Electrostatic “hot-spots”, generated by clusters of like-charged residues, are frequent in nature, since they provide rapid association of biomolecules satisfying the need for speed. However, there is a penalty paid in the form of a loss in local protein stability, since the presence of clusters of like-charged residues can result in numerous unfavorable intramolecular Coulombic interactions, as has been shown for the case of barnase-barstar [27]. Fersht and coworkers have proposed that nature often selects for function over stability [27], as is suggested by the presence of electrostatic “hot-spots” on many proteins. In the case of human C3d, electrostatic “hot-spots” have evolved surrounding the thioester bond and CR2 binding site, which when combined accelerate the formation of the B-cell co-receptor complex, a link between innate and adaptive immunity. Despite the lack of electrostatic “hot-spots” on human CR2, the excessively positive nature of CR2 has likely been a driving force in the evolution of the electrostatic “hot-spot” on the acidic face of C3d (Figure 6). Similarly, the acidic nature of the CR2 binding site on C3d is likely the reason why CR2 of higher species has the highest net charge, as is seen in chimpanzees and humans (Figure 2). Furthermore, we propose that the more even distribution of charged residues observed on human CR2(CCP1-2) is sufficient given the mono-functionality of CR2(CCP1-2), while the electrostatic “hot-spots” of C3d have evolved to optimize the dual-functionality of C3d.

The C3d-CR2 interaction greatly improves the immune response to an antigen, and as a result has been selected by nature as a target for immune evasion [18]–[20]. Structural evidence has shown that virulence factors of *Staphylococcus aureus* target the electrostatic “hot-spots” of human C3d (Supporting Figure S2). For example, *Staphylococcus aureus* secretes the highly cationic virulence factors EfbC and Ehp, which take advantage of the CR2 electrostatic “hot-spot” through the use of long-range, as well as short-range, electrostatic interactions (Supporting Figure S2A) [18]. Additionally, domain IV of the Staphylococcal immunoglobulin-binding protein (Sbi) targets the thioester side electrostatic “hot-spot” of C3d (Supporting Figure S2B), and in conjunction with Sbi domain III results in futile consumption of C3 through the formation of covalent adducts [19]. The electrostatic nature and binding sites of the *Staphylococcus aureus* virulence factors is further evidence for the key role of electrostatics in the function and evolution of complement C3d.

In general, electrostatic calculations, such as those presented here, can provide insight into the evolution of protein function; however, electrostatic similarity alone cannot be used to derive evolutionary (phylogenetic) relationships. Due to the long-range nature of electrostatics, proteins with very different sequences can result in very similar electrostatic potentials, such is the case when comparing the human and amphioxus C3d homologues. The similarity observed in the electrostatic potentials/”hot-spots” of human and amphioxus C3d, suggests that both homologues have evolved to have similar function, and we even predict that amphioxus C3d could bind human CR2; that said, these electrostatically similar proteins have resulted from different sets of evolutionary pressures and our current understanding would suggest that there is not an amphioxus CR2 homologue. In this study, our perturbation map analysis is intended to complement standard sequence-based analysis by providing insights into the evolution of function according to electrostatics-based arguments. When comparing the homologues of complement C3d, a weak CR2 “hot-spot” doesn't necessarily imply an absence of the C3d-CR2 interaction, but implies a less optimized interaction, specifically with respect to electrostatics. Therefore, based on our analysis we cannot conclude which homologues of C3d interact with a CR2 homologue, but we have identified the onset of a new role of charge/electrostatics in the function of C3d after the divergence of jawless fish. We propose that this new role of charge corresponds with the appearance of the first multi-functional homologue of C3d. It should be noted that conserved electrostatic potential is not necessarily indicative of a conserved electrostatic “hot-spot”, as can be seen when comparing the homologues of C3d (Figures 3A and 5). Our novel methods based on perturbation maps identified the electrostatic “hot-spots” of C3d and have potential utility in the identification of functional sites of other highly-charged biomolecular systems, as well as in drug design.

## Methods

All calculations for human C3d, as well as all homology modeling, was based on the crystal structure of unbound human C3d (PDB Code: 1C3D) [10]. The sequence for human complement C3d was extracted from 1C3D and was used as a Blast query to identify C3d homologue sequences from the UniProt database [28]. The 23 C3d homologues were selected to optimize the range of sequence similarity when compared to human C3d (∼37–85% identity with human), but were chosen while keeping in mind that ∼40% similarity is needed to ensure accuracy when performing homology modeling (Supporting Table S1). Similarly, a crystal structure of unbound CR2(CCP1-2) (PDB Code: 1LY2) was used for all CR2 related homology modeling and electrostatics calculations. The sequences for the 4 CR2 homologues were obtained from the NCBI UniGene Project, and to the best of our knowledge represent all known CR2 sequences to date.

As an initial comparison, a multiple sequence alignment comparing the 24 C3d homologues was generated using MUSCLE [29] and Bio3D [30] (Supporting Figure S6). The resulting alignment was used to populate a pairwise sequence identity matrix comparing the 24 C3d homologues. A second matrix comparing pairwise charge similarity was also generated by identifying the number of amino acid positions with a like charged amino acid for each pair of sequences, meaning the number of positions where both sequences have either K/R or D/E. The generated similarity matrices were used as input for hierarchical clustering using the R statistical language [31], and the results were visualized using heatmaps with dendrograms. The functional regions for sequence clustering were defined based on the x-coordinates of the residues as illustrated by Supporting Figure S7.

In this study, homology modeling was utilized to generate structures for the 23 non-human C3d homologues based on 1C3D, as well as for the 3 non-human homologues of CR2 (details in Text S1). Additionally, perturbed structures of the 24 C3d homologues were generated using a combination of alanine-scan mutagenesis and a molecular dynamics simulation (details in Text S1). All electrostatic potential calculations were performed using APBS [32], based on a grid with 129×129×129 grid points and lengths of 98 Å×116 Å×116 Å and 102 Å×78 Å×116 Å for C3d and CR2, respectively. The solvent environment was represented by a dielectric constant of 78.57 with a counterion concentration of 0 mM, while the protein dielectric constant was 20. An ionic strength of 0 mM was selected, since in previous studies [23] involving similar alanine-scan perturbations of barnase-barstar and electrostatic clustering, we found that 0 mM calculations performed better when considering the effects of dynamics. Additionally, the same study showed that electrostatic free energies calculated at 0 and 150 mM ionic strength had near identical correlations with experiment. Similarly, we have also shown that when considering alanine mutations of ionizable amino acids for highly charged proteins, that correlations between calculated and experimental free energies improved using a protein dielectric of 20 rather than the typical value of 2 [33], which is the motivation for selecting a protein dielectric of 20 for this study. A probe serve with a radius of 1.4 Å was used to define the dielectric boundary. Protonation states of ionizable amino acids were assigned according to model pK_a_ values at a pH of 7.4. Each electrostatic potential calculation was centered on either 1C3D or 1LY2 to ensure proper alignment of electrostatic potentials prior to similarity calculations. For each set of electrostatic potentials, cumulative distributions of electrostatic similarity (ESI) were calculated using the AESOP framework, according to the following expression:
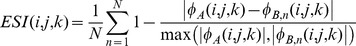
Here, *φ_A_* represents the electrostatic potential to which all other potentials are compared (parent), while *φ_B,n_* represents the *N* members of the family of electrostatic potentials to be compared. The *ESI* is calculated at each grid point *(i,j,k)*, and normalized by *N*, the number of electrostatic potentials comparisons. This measure of electrostatic similarity only describes the similarity of the electrostatic potential of a set of proteins to one particular protein at a given grid point. For example, the ESI distribution for the C3d homologues (Figure 3A) was calculated by comparing the electrostatic potentials of the 23 homology models to the electrostatic potential of 1C3D. The surface projections of electrostatic similarity were generated using UCSF Chimera [34].

## Supporting Information

Figure S1 Complement regulators and receptors bind the acidic “hot-spot” of complement C3d. Cumulative electrostatic similarity distribution for 24 homologues projected onto the surface of human C3d (same as Figure 3A) [blue – green – red; low to high similarity] with host ligands superimposed. Ribbon representations are used for the host ligands: FH 4 – white (PDB: 2WII); FH 19/20 – gray (PDB: 2XQW); CR2 – black (PDB: 3OED). Two rotations of C3d (180 degrees about the y-axis) are provided to show the two electrostatic “hot-spots”: (A) CR2-face and (B) thioester-face.
(TIFF)

Figure S2 Pathogenic inhibitors of the complement system target the conserved electrostatic “hot-spots” of complement C3d. Cumulative electrostatic similarity distribution for 24 homologues projected onto the surface of human C3d (same as Figure 2B) [blue – green – red; low to high similarity] with *S. aureus* virulence factors superimposed. Ribbon representations are used for the *S. aureus* virulence factors: Ehp – white (PDB: 2NOJ); Efb-C – gray (PDB: 2GOX); Sbi – black (PDB: 2WY7). Two rotations of C3d (180 degrees about the y-axis) are provided to show the two electrostatic “hot-spots”: (A) CR2-face and (B) thioester-face.
(TIFF)

Figure S3 Electrostatic similarity distributions for perturbed human CR2 structures. Perturbation maps based on a theoretical alanine scan, consisting of 24 charged residue to alanine mutations. Color scheme is: blue – green – red; low to high similarity, corresponding to ESI values of: (A) 0.5–0.7–0.9 and (B) 0.47–0.48–0.49.
(TIF)

Figure S4 Sequence and charge clustering of C3d homologues using whole sequences. Dendrograms with distance matrix heatmaps illustrate sequence clustering based on: (A) percent identity and (B) number of positions with the same charge. Net charge of each sequence is provided in parentheses.
(TIFF)

Figure S5 Sequence similarity clustering for the two sides of C3d. Dendrograms with distance matrix heatmaps illustrate clustering of the 24 C3d homologues based on percent identities within the two functional regions as defined by Supporting Figure 6: (A) CR2-face and (B) thioester-face. Net charge of each homologue is provided in parentheses.
(TIFF)

Figure S6 Multiple sequence alignment of 24 C3d homologues. A consensus sequence, as well as bars indicating conservation and charge variation per position, are included for comparison. The Clustal X coloring scheme (as implemented in UCSF Chimera), which is dependent on amino acid and conservation, was used to color the sequences.
(TIF)

Figure S7 Illustration of the two functional regions used for sequence analysis of complement C3d. Residues were assigned to the two regions according to their x-coordinates. The CR2-face (colored in red) includes residues that contain at least one atom with an x-coordinate ≤ (mean(x) −5 Å), while thioester face includes residues that contain at least one atom with an x-coordinate > (mean(x)+5 Å).
(TIFF)

Table S1 List of complement C3d homologues with UniProt accession ID, human C3d percent identity, and net charge.
(TIFF)

Text S1 Details of homology modeling and molecular dynamics simulations.
(DOCX)
