# Supplementary figures and images for: The Two Sides of Complement C3d: Evolution of Electrostatics in a Link between Innate and Adaptive Immunity
Source: PLoS Comput Biol. 2012 Dec 27;8(12):e1002840. doi: 10.1371/journal.pcbi.1002840 (PMC3531323; doi:10.1371/journal.pcbi.1002840)

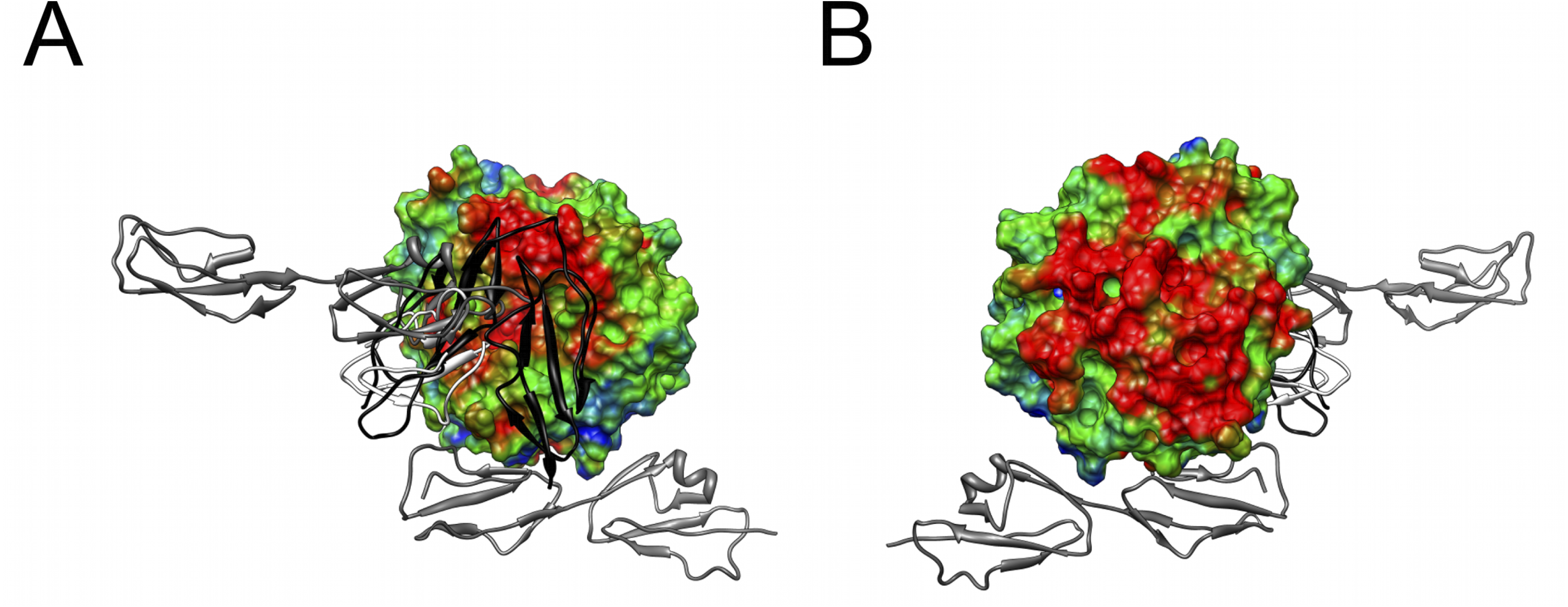

Supplement: Figure S1 — Complement regulators and receptors bind the acidic “hot-spot” of complement C3d. Cumulative electrostatic similarity distribution for 24 homologues projected onto the surface of human C3d (same as Figure 3A) [blue – green – red; low to high similarity] with host ligands superimposed. Ribbon representations are used for the host ligands: FH 4 – white (PDB: 2WII); FH 19/20 – gray (PDB: 2XQW); CR2 – black (PDB: 3OED). Two rotations of C3d (180 degrees about the y-axis) are provided to show the two electrostatic “hot-spots”: (A) CR2-face and (B) thioester-face. (TIFF) [file pcbi.1002840.s001.tiff]

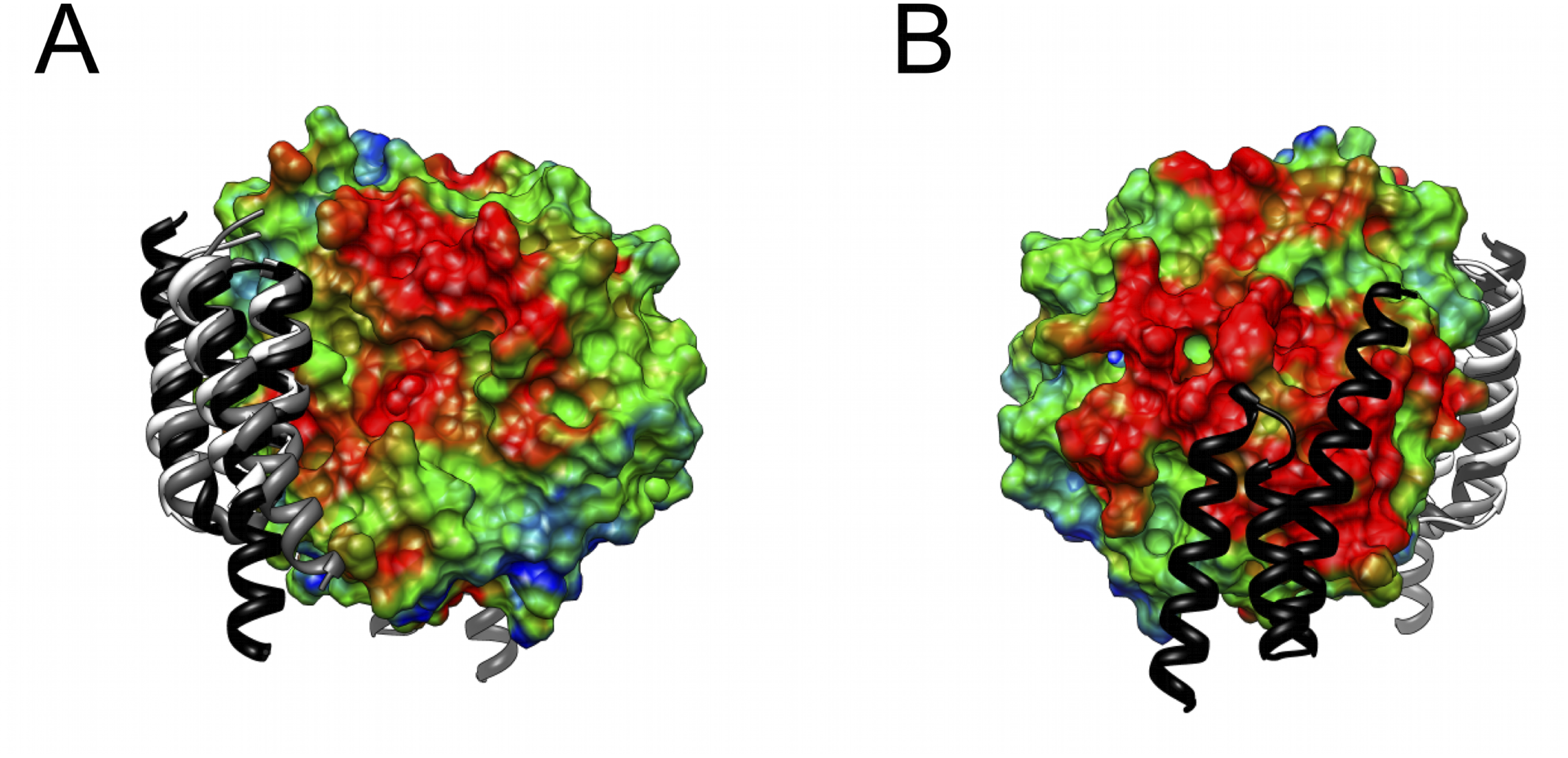

Supplement: Figure S2 — Pathogenic inhibitors of the complement system target the conserved electrostatic “hot-spots” of complement C3d. Cumulative electrostatic similarity distribution for 24 homologues projected onto the surface of human C3d (same as Figure 2B) [blue – green – red; low to high similarity] with S. aureus virulence factors superimposed. Ribbon representations are used for the S. aureus virulence factors: Ehp – white (PDB: 2NOJ); Efb-C – gray (PDB: 2GOX); Sbi – black (PDB: 2WY7). Two rotations of C3d (180 degrees about the y-axis) are provided to show the two electrostatic “hot-spots”: (A) CR2-face and (B) thioester-face. (TIFF) [file pcbi.1002840.s002.tiff]

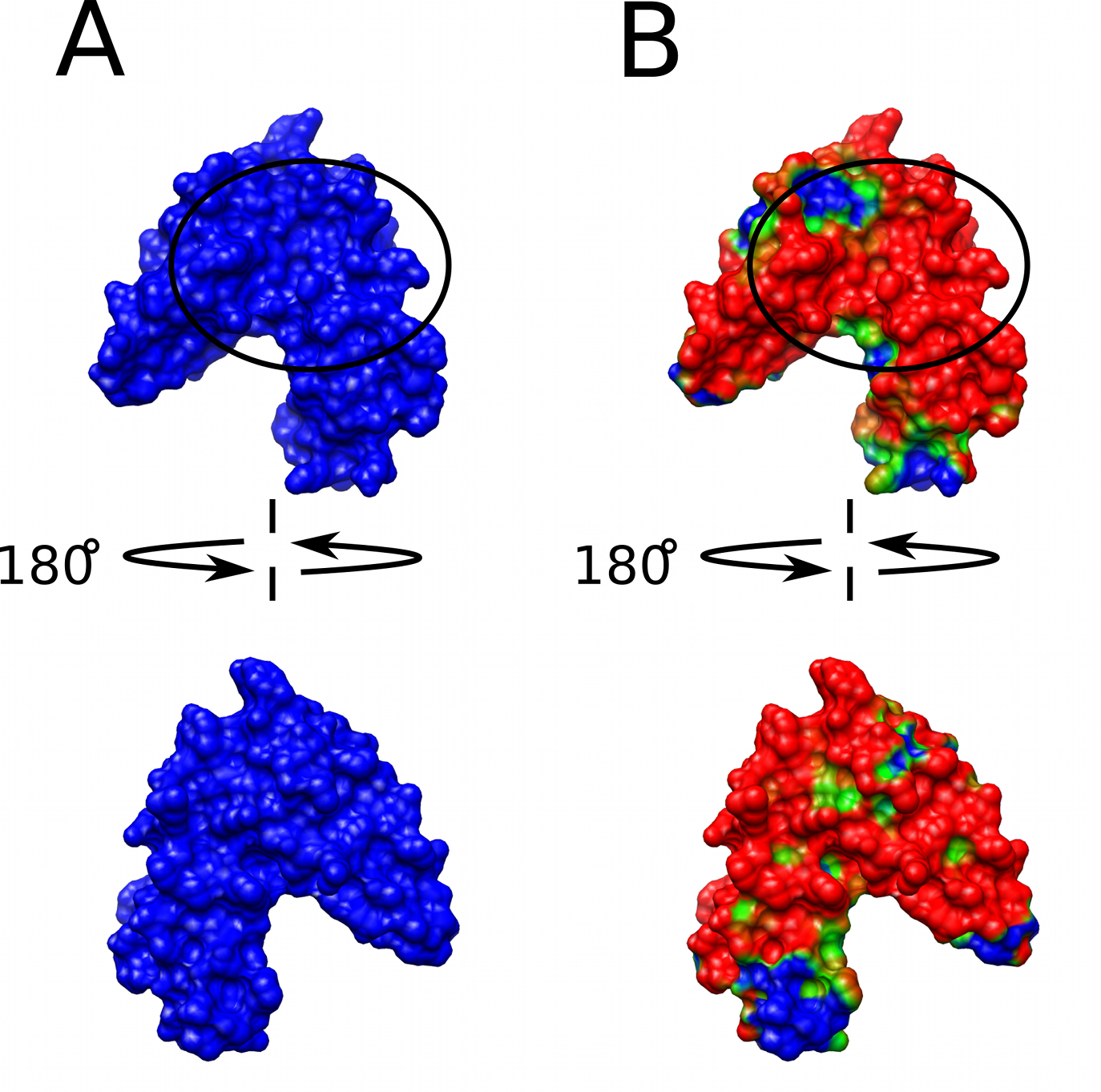

Supplement: Figure S3 — Electrostatic similarity distributions for perturbed human CR2 structures. Perturbation maps based on a theoretical alanine scan, consisting of 24 charged residue to alanine mutations. Color scheme is: blue – green – red; low to high similarity, corresponding to ESI values of: (A) 0.5–0.7–0.9 and (B) 0.47–0.48–0.49. (TIF) [file pcbi.1002840.s003.tif]

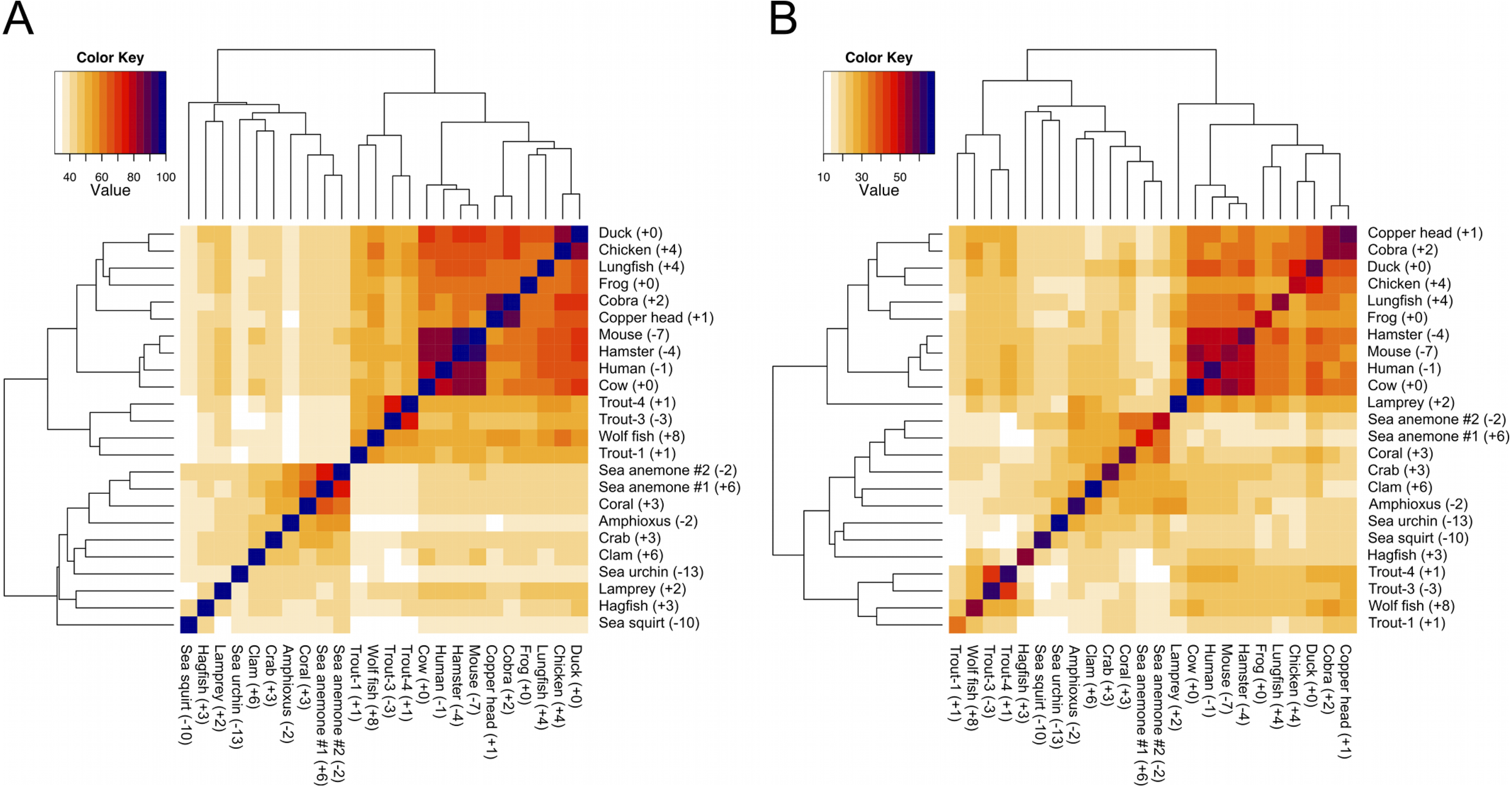

Supplement: Figure S4 — Sequence and charge clustering of C3d homologues using whole sequences. Dendrograms with distance matrix heatmaps illustrate sequence clustering based on: (A) percent identity and (B) number of positions with the same charge. Net charge of each sequence is provided in parentheses. (TIFF) [file pcbi.1002840.s004.tiff]

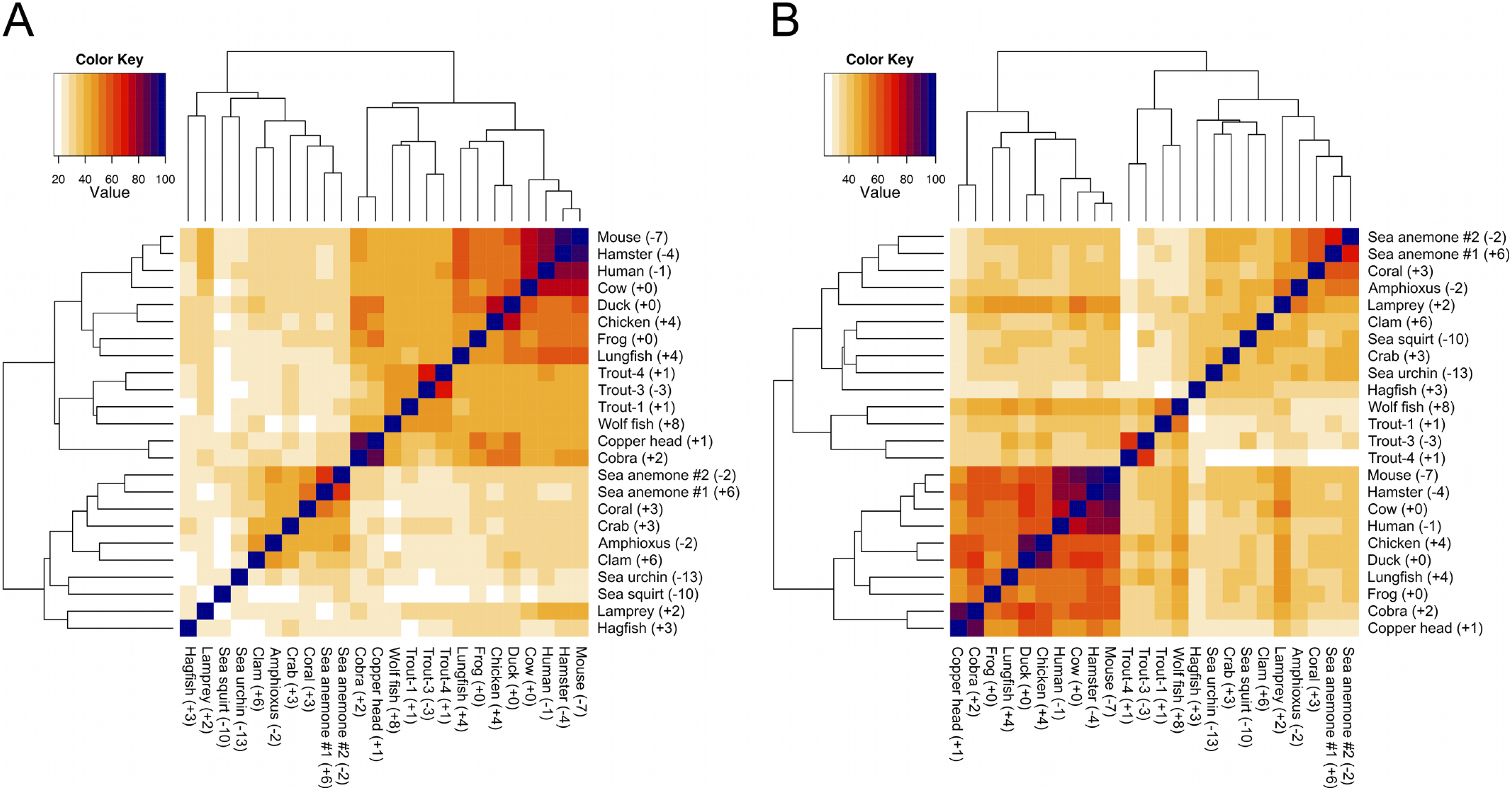

Supplement: Figure S5 — Sequence similarity clustering for the two sides of C3d. Dendrograms with distance matrix heatmaps illustrate clustering of the 24 C3d homologues based on percent identities within the two functional regions as defined by Supporting Figure 6: (A) CR2-face and (B) thioester-face. Net charge of each homologue is provided in parentheses. (TIFF) [file pcbi.1002840.s005.tiff]

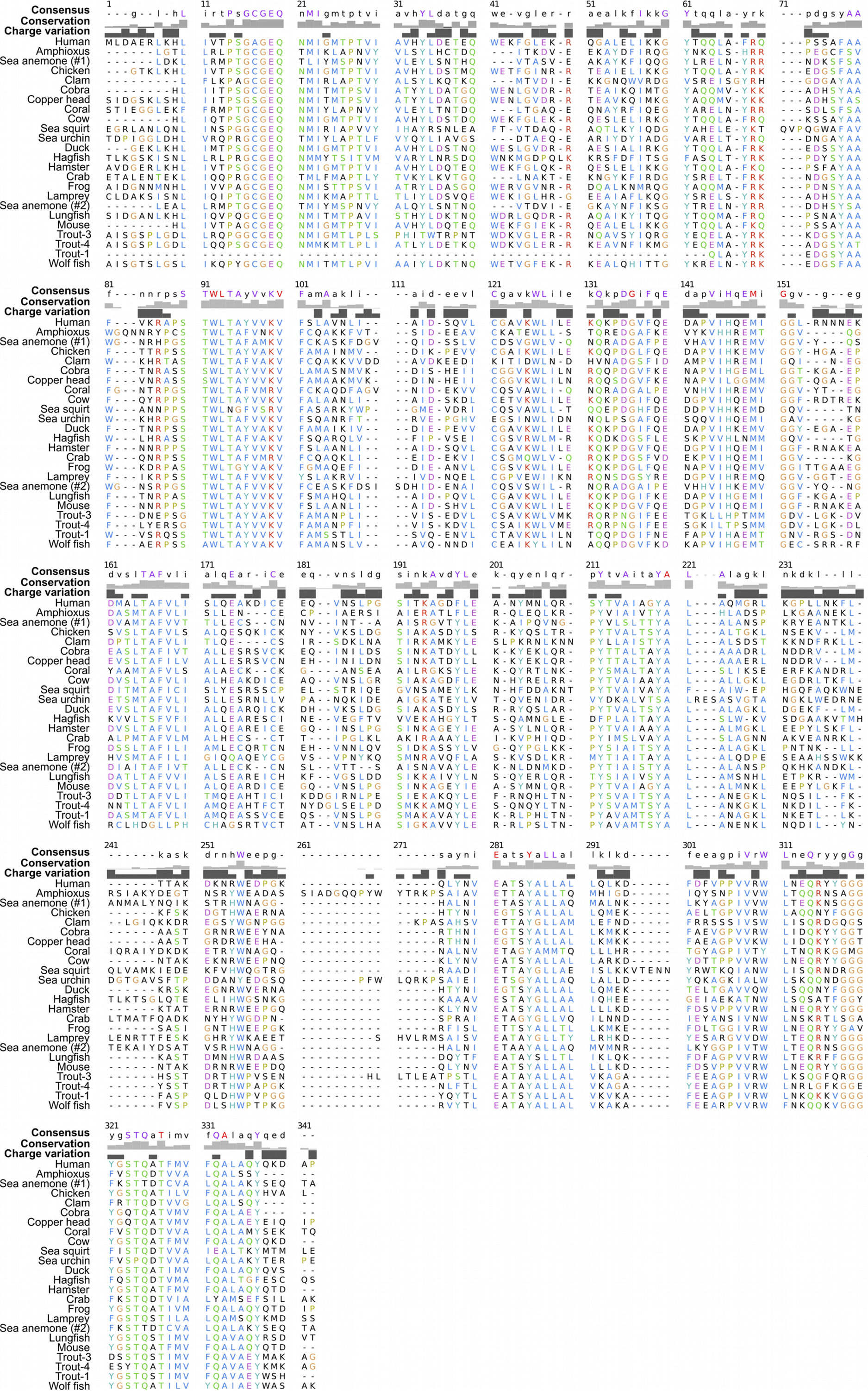

Supplement: Figure S6 — Multiple sequence alignment of 24 C3d homologues. A consensus sequence, as well as bars indicating conservation and charge variation per position, are included for comparison. The Clustal X coloring scheme (as implemented in UCSF Chimera), which is dependent on amino acid and conservation, was used to color the sequences. (TIF) [file pcbi.1002840.s006.tif]

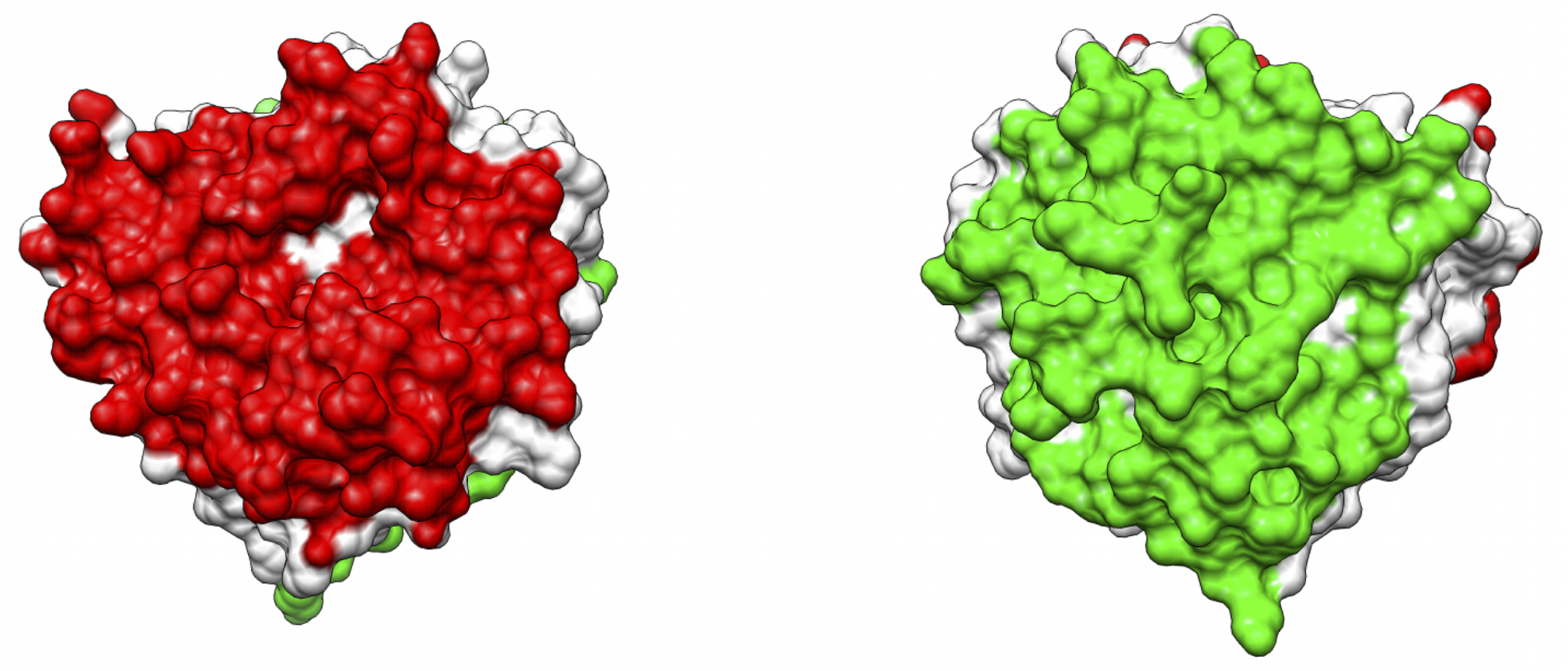

Supplement: Figure S7 — Illustration of the two functional regions used for sequence analysis of complement C3d. Residues were assigned to the two regions according to their x-coordinates. The CR2-face (colored in red) includes residues that contain at least one atom with an x-coordinate ≤ (mean(x) −5 Å), while thioester face includes residues that contain at least one atom with an x-coordinate > (mean(x)+5 Å). (TIFF) [file pcbi.1002840.s007.tiff]

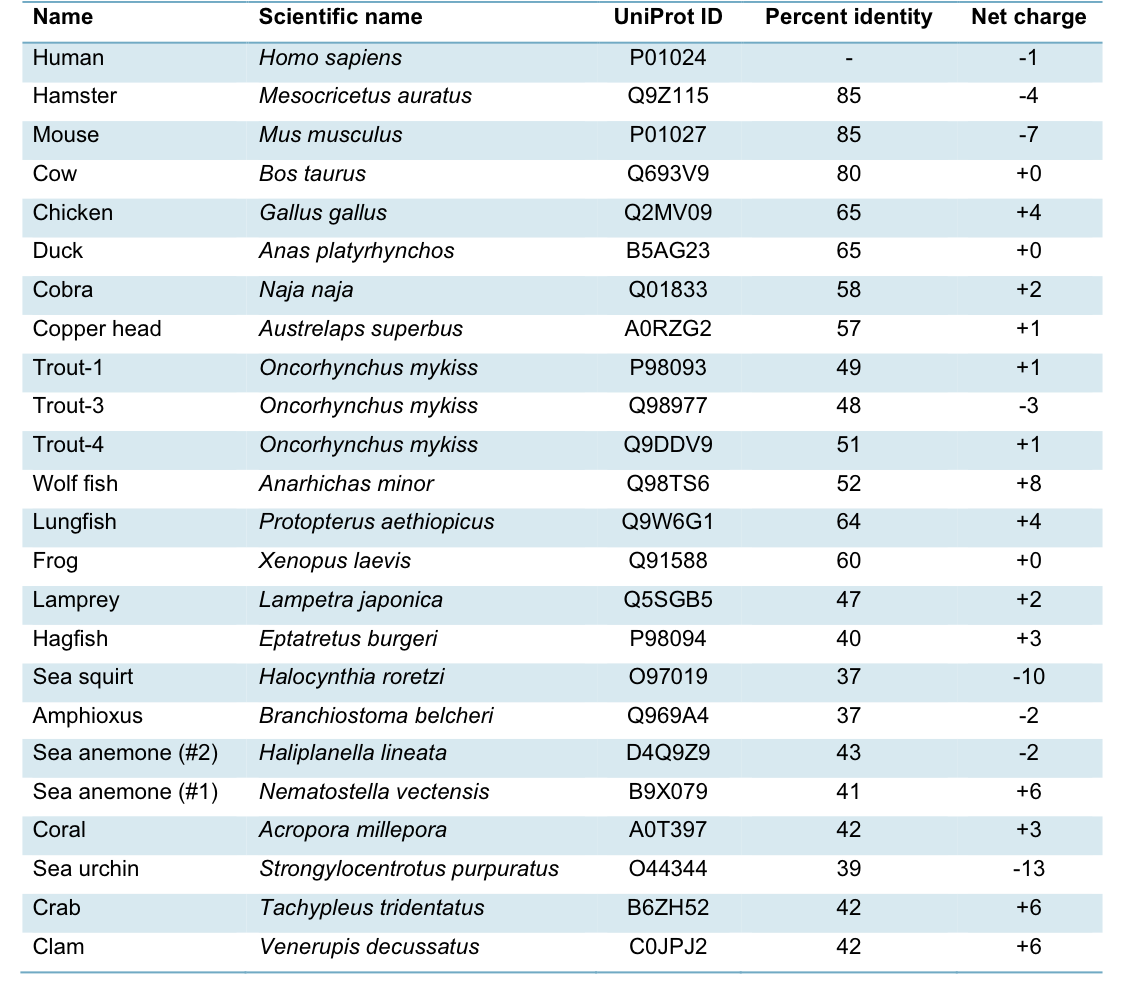

Supplement: Table S1 — List of complement C3d homologues with UniProt accession ID, human C3d percent identity, and net charge. (TIFF) [file pcbi.1002840.s008.tiff]
