# Supplementary material for: The Two Sides of Complement C3d: Evolution of Electrostatics in a Link between Innate and Adaptive Immunity
Source: PLoS Comput Biol. 2012 Dec 27;8(12):e1002840. doi: 10.1371/journal.pcbi.1002840 (PMC3531323; doi:10.1371/journal.pcbi.1002840)
Supplement: Text S1 — Details of homology modeling and molecular dynamics simulations. (DOCX) [file pcbi.1002840.s009.docx]

The two sides of complement C3d: Evolution of electrostatics in a link between innate and adaptive immunity.

Supporting Information

(7 Figures, 1 Table, and Extended Methods)

Chris A. Kieslich and Dimitrios Morikis*

Department of Bioengineering, University of California, Riverside; Riverside, CA 92521

**Text S1.** Details of homology modeling and molecular dynamics simulations.

Homology modeling and structure preparation:

Homology modeling was performed using the Automodel module of Modeller 9v9 (1) to produce structures for the 23 additional homologues of complement C3d and 3 non-human homologues of CR2. Crystal structures of unbound human complement C3d (PDB Code: 1C3D) and CR2 (PDB Code: 1LY2) were used as the template structure. Sequence alignments between human C3d and the 23 C3d homologues, as well as CR2 and its 3 homologues, were generated using MUSCLE in conjunction with the R package Bio3D, and supplied as input to Modeller. Each homology model was subsequently superimposed onto 1C3D using Bio3D. The generated models were then prepared using PDB2PQR (2), in order to add atomic radii and partial charges according to the PARSE (3) forcefield to the coordinate files. Alanine-scan mutagenesis was performed using the coordinates of the two crystal structures and generated homology models as parent structures and applying the AESOP computational framework, Analysis of Electrostatic Similarities of Proteins (4), which truncates each ionizable residue (Arg, Lys, His, Glu, and Asp) to alanine, one at a time. No structural relaxation was considered after each mutation.

Molecular dynamics simulation:

A 20-ns explicit-solvent molecular dynamics simulation was configured using VMD (5) and performed using NAMD (6). The crystal structure of human C3d (1C3D) was placed in a 82 Å × 82 Å × 82 Å water box with NaCl ions at 150 mM ion strength. The simulation was performed using the NPT ensemble with Langevin temperature and pressure control, at 300 K. The system was initially minimized using 1000 steps of energy minimization, to remove unfavorable torsions and contacts. The system was further relaxed by constraining the protein while allowing the solvent to equilibrate for 100 ps. Constraints were reduced linearly at 100 ps intervals, resulting in a total of 1 ns of equilibration. The simulation was performed using a 2 fs time step with rigid bonds to hydrogen imposed by SHAKE.

1. Marti-Renom M et al. (2000) Comparative protein structure modeling of genes and genomes. *Annu Rev Bioph Biom* 29:291–325.

2. Dolinsky T, Nielsen J, McCammon J, Baker N (2004) PDB2PQR: an automated pipeline for the setup of Poisson-Boltzmann electrostatics calculations. *Nucleic Acids Res* 32:W665–W667.

3. Sitkoff D, Sharp K, Honig B (1994) Accurate calculation of hydration free energies using macroscopic solvent models. *J Phys Chem* 98:1978–1988.

4. Kieslich CA, Gorham RD, Morikis D (2011) Is the rigid-body assumption reasonable? Insights into the effects of dynamics on the electrostatic analysis of barnase-barstar. *J Non-Cryst Solids* 357:707–716.

5. Humphrey W, Dalke A, Schulten K (1996) VMD: Visual molecular dynamics. *J Mol Graphics* 14:33–38.

6. Phillips J et al. (2005) Scalable molecular dynamics with NAMD. *J Comput Chem* 26:1781–1802.
